# Supplementary figures and images for: The Effects of Nature Exposure Therapies on Stress, Depression, and Anxiety Levels: A Systematic Review
Source: Eur J Investig Health Psychol Educ. 2024 Mar 7;14(3):609–22. doi: 10.3390/ejihpe14030040 (PMC10969128; doi:10.3390/ejihpe14030040)

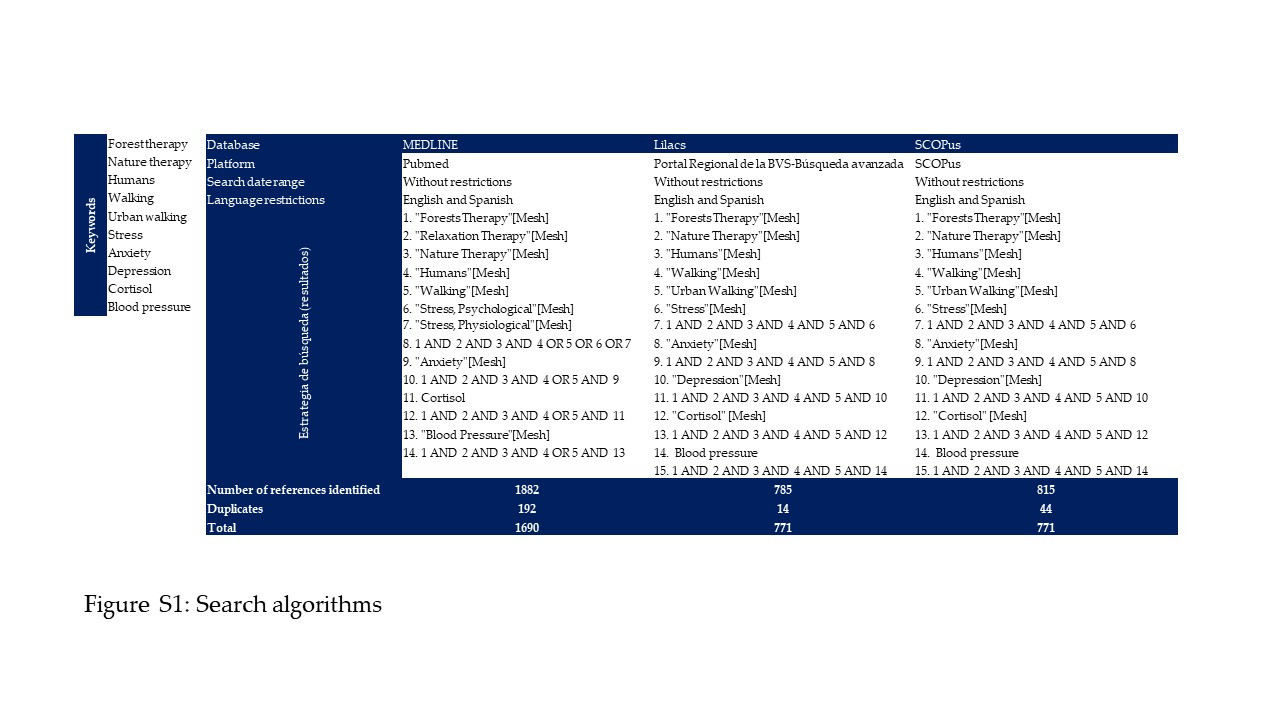

Supplement: Supplementary file 1 [file ejihpe-14-00040-s001.zip › Figure S1.tif]

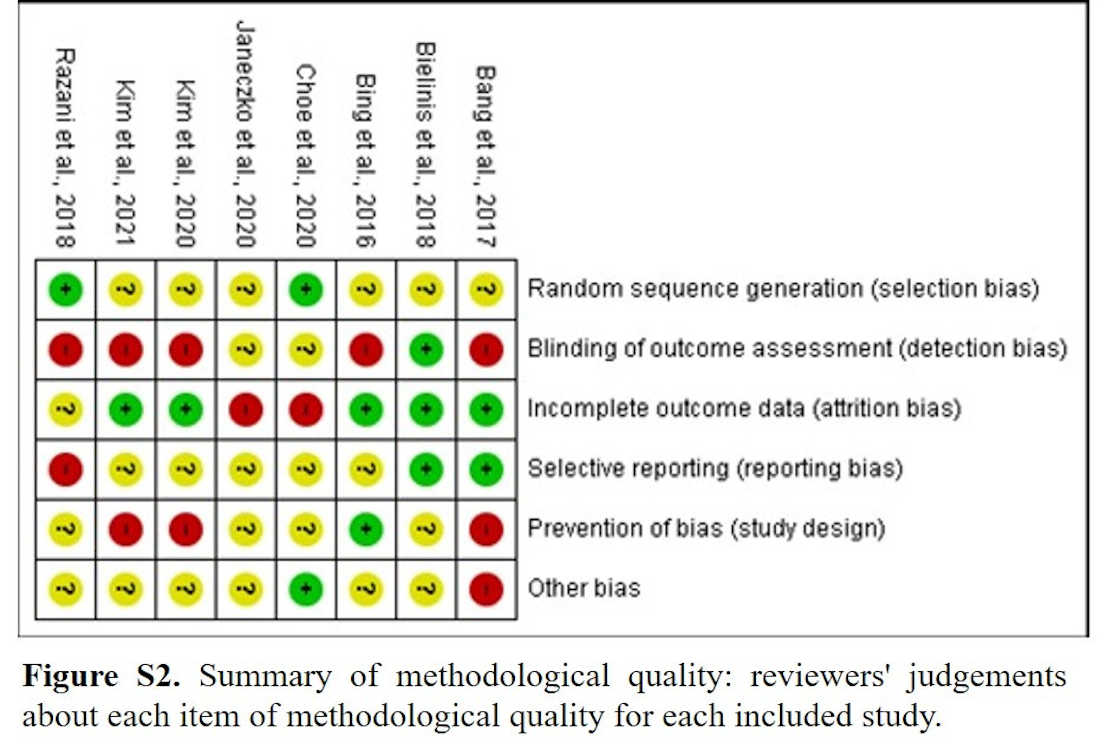

Supplement: Supplementary file 1 [file ejihpe-14-00040-s001.zip › Figure S2.tif]

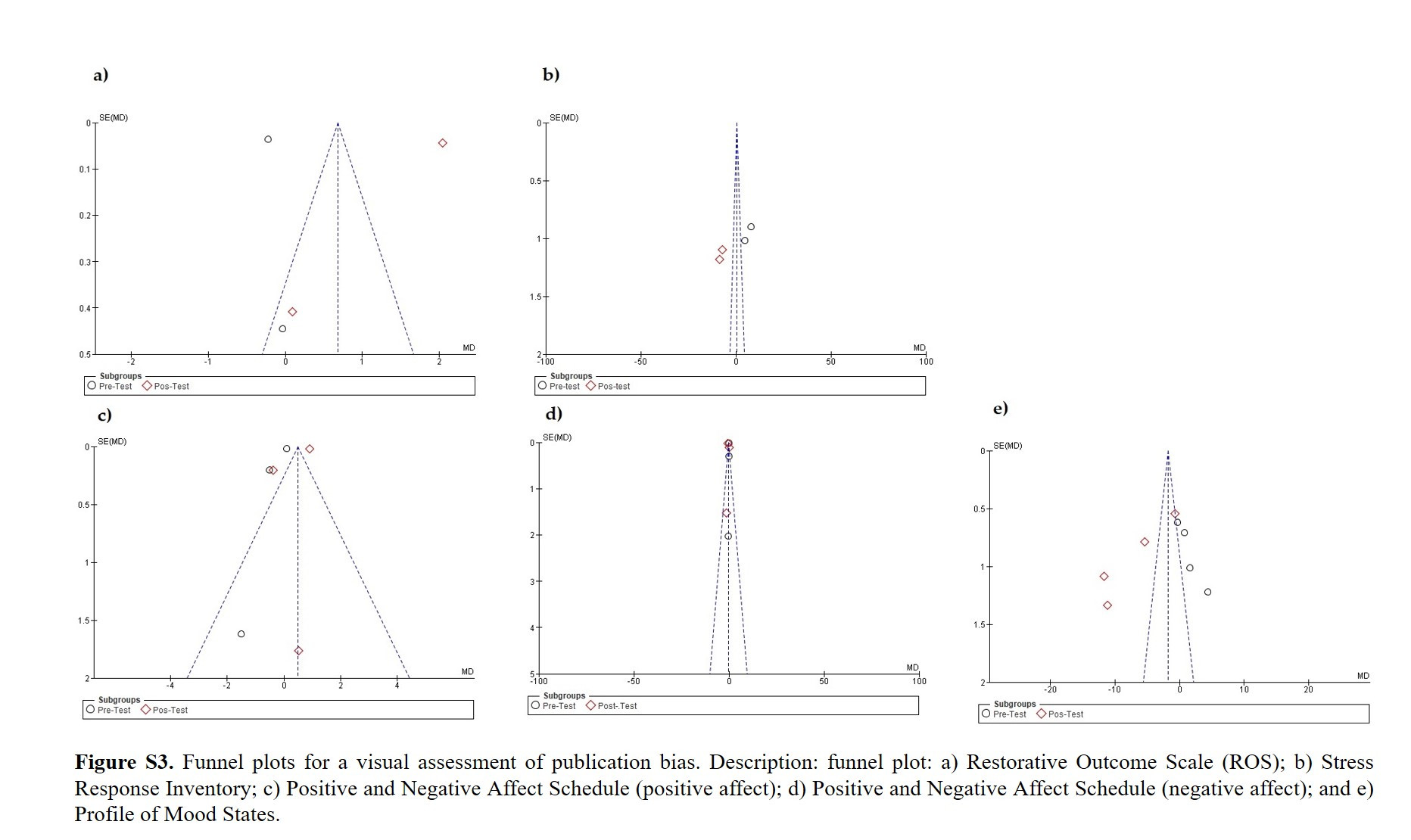

Supplement: Supplementary file 1 [file ejihpe-14-00040-s001.zip › Figure S3.tif]
